# Self-Biased Bidomain LiNbO_3_/Ni/Metglas Magnetoelectric Current Sensor

**DOI:** 10.3390/s20247142

**Published:** 2020-12-13

**Authors:** Mirza I. Bichurin, Roman V. Petrov, Viktor S. Leontiev, Oleg V. Sokolov, Andrei V. Turutin, Victor V. Kuts, Ilya V. Kubasov, Alexander M. Kislyuk, Alexander A. Temirov, Mikhail D. Malinkovich, Yuriy N. Parkhomenko

**Affiliations:** 1 Department of Design and Technology of Radioequipment, Yaroslav-the-Wise Novgorod State University, ul. B. St. Petersburgskaya, 41, 173003 Veliky Novgorod, Russia; Roman.Petrov@novsu.ru (R.V.P.); s181607@std.novsu.ru (V.S.L.); Oleg.Sokolov@novsu.ru (O.V.S.); 2 Department of Materials Science of Semiconductors and Dielectrics, National University of Science and Technology MISiS, Leninskiy Prospekt 4, 119049 Moscow, Russia; a.turutin@misis.ru (A.V.T.); Vkuts@misis.ru (V.V.K.); ilya.kubasov@misis.ru (I.V.K.); kisliuk.am@misis.ru (A.M.K.); temirov.alex@yandex.ru (A.A.T.); malinkovich@yandex.ru (M.D.M.); parkh@rambler.ru (Y.N.P.); 3 Department of Physics and I3N, University of Aveiro, 3810-193 Aveiro, Portugal

**Keywords:** magnetoelectric effect, magnetoelectric gradient structure, bidomain lithium niobate, magnetoelectric sensor, current sensor

## Abstract

The article is devoted to the theoretical and experimental study of a magnetoelectric (ME) current sensor based on a gradient structure. It is known that the use of gradient structures in magnetostrictive-piezoelectric composites makes it possible to create a self-biased structure by replacing an external magnetic field with an internal one, which significantly reduces the weight, power consumption and dimensions of the device. Current sensors based on a gradient bidomain structure LiNbO_3_ (LN)/Ni/Metglas with the following layer thicknesses: lithium niobate—500 μm, nickel—10 μm, Metglas—29 μm, operate on a linear section of the working characteristic and do not require the bias magnetic field. The main characteristics of a contactless ME current sensor: its current range measures up to 10 A, it has a sensitivity of 0.9 V/A, its current consumption is not more than 2.5 mA, and its linearity is maintained to an accuracy of 99.8%. Some additional advantages of a bidomain lithium niobate-based current sensor are the increased sensitivity of the device due to the use of the bending mode in the electromechanical resonance region and the absence of a lead component in the device.

## 1. Introduction

Electric current sensors are an important type of electronic device that are widely used from nanoelectronic systems to complex electronic and robotic complexes, where the range of measured currents is enormous, from 10^−6^ A (leakage currents) to hundreds of A. Currently, there are a large number of current sensors operating on various physical principles; traditionally, electric current sensors use either Hall effect devices or magnetoresistive devices [1,2,3]. However, due to their low sensitivity, Hall sensors suffer from a low Hall voltage when measuring low currents, which requires the use of a highly accurate signal processing system, and the same problem occurs with magnetoresistive sensors, which leads to an increase in the cost of these sensors. A significant breakthrough in measuring technology is the idea of using the ME effect to build various sensors. These ideas arose among researchers after the appearance of the fundamental works by Harshe et al. [4,5], in which the theory of the ME effect in layered and bulk magnetostrictive-piezoelectric composites based on CFO-PZT and CFO-BTO was supported by experimental results. It was shown that when the ME composite is exposed to external constant and alternating magnetic fields, as a result of internal magnetostrictive and piezoelectric interactions, an electric voltage is induced on the composite. It becomes obvious that in this way it is possible to measure the constant or alternating magnetic field acting on the composite, and this was confirmed by the appearance of the first work on the ME magnetic field sensor presented at the MEIPIC-4 International Conference [6], in which a constant magnetic field sensor based on a bulk composite from ferrites (YIG or NFO) and PZT ceramics was discussed. The new developments in the investigation of ME composite materials in recent years have opened the way to create low-cost commercial ultra-sensitive magnetic and current sensors operating at room temperature [7,8,9,10,11,12,13,14,15,16,17,18,19,20,21].

Since the magnetic field and current are interrelated parameters, soon, naturally, an extensive number of articles [20,22,23,24,25,26,27,28,29,30,31,32] and reviews [33,34] on ME current sensors appeared. Below we give a brief overview of the most famous works in this area. The first sensor designs were contactless ring structures with a current conductor running through the center. In the first work, S. Dong et al. [22] presented a current sensor based on a quasi-ring-type laminate composite consisting of a piezoelectric PZN-4,5PZT ring and two magnetostrictive Terfenol-D rings. This sensor showed a very large ME voltage coefficient of 2.2 V/Oe (5.5 V/(cm·Oe)) and promising capabilities to detect AC currents as small as 10^−7^ A, and/or a vortex magnetic field as small as 6∙10^−12^ T over the frequency range of 0.5 < *f* < 105 Hz. Other types of ring current sensors were described in [25,26,29]. Current sensors [25,26] have operated in vortex magnetic field detection mode and consisted of a ring-shaped polarized PZT piezoelectric ceramic ring bonded between magnetized epoxy-bonded Terfenol-D short-fiber/NdFeB magnet magnetostrictive composite rings. The best of these sensors showed high and linear current sensitivities and magnetoelectric voltage coefficients of 15–17 mV/A and 22–26 mV/Oe in the nonresonance frequency range of 1 Hz to 50 kHz, and of 185 mV/A and 277 mV/Oe at the fundamental shape resonance of 96 kHz, for current and vortex magnetic field levels up to 10 A and 6.7 Oe. Using the epoxy-bonded Terfenol-D short-fiber/NdFeB magnet magnetostrictive composite rings allowed increasing the sensitivity of these passive sensors. Zhang S. et al. [29] added a Rosen-type PZT piezoelectric transformer to the ring structure; they had the sensor work in two modes: current sensing mode and current transduction mode. The current transduction mode gave 6.4 times higher resonance sensitivity, reaching 1000 mV/A at 62 kHz, while the current sensing mode had 157 mV/A at the same frequency. The next group of current sensors had a standard three-layer design, consisting of two layers of longitudinally magnetized Terfenol-D and one layer of transversely polarized PZT [26], or two layers of transversely magnetized Terfenol-D and transversely polarized PZT, i.e., T-T mode [31]. A self-powered current sensor consisting of the magnetostrictive/piezoelectric laminate composite and the high-permeability nanocrystalline alloys was presented in [27]. The main advantages of this current sensor are its large dynamic range and its ability to measure currents accurately. The dynamic range of this sensor is from 0.01 to 150 A, and a small electric current step-change of 0.01 A can be clearly distinguished at the power-line frequency of 50 Hz. Yu X. et al. suggested a slice-type magnetoelectric laminated current sensor [30] and noted the convenience and practicality of this sensor when compared to ring sensors. The sensor was installed on the wire with the measured current by means of epoxy glue. The current sensitivity was 1.03 mV/mA over input currents ranging from 15 mA to 2.1 A. A stable output voltage over a frequency range from 20 to 5 kHz was attained by slice-type. The current sensor in [31] was used to measure direct current. Under resonant AC drive, a stable induced voltage, more than 9 V, changes with the variation of the measured DC current over the range of 0~500 A, indicating that this sensor is applicable for measuring high DC currents. The DC current sensitivity over the range of 0~500 A is 2.42 mV/A with 1 A AC current at the resonant frequency 84.9 kHz. The use of the Terfenol-D hard magnetic magnet in current sensors required the use of large bias fields (100–500 Oe) and, accordingly, large permanent magnets. Therefore, in the further development of current sensors, magnetically soft materials of the Metglas type [28,32] began to be used more often, which made it possible to reduce the bias fields to 5–20 Oe. In [28] Lu C. et al. presented the sensor on a two-layer Metglas-PZT composite in the bending mode. By adding a 1.3 g tip mass at the free end of Metglas/PZT, the resonant frequency of Metglas/PZT with the number of Metglas layers L = 4 can be adjusted to 50 Hz, where the V_0_ is 211 mV at H_dc_ = 16 Oe. The sensor showed excellent linearity and large current sensitivity (114.2 mV/A) when measuring low-frequency alternating magnetic fields of 50 Hz. Another current sensor [32] based on Metglas-PZT demonstrated operation in two modes: non-resonant (500 Hz) and resonant (168 kHz). The bias field was 10 Oe. The characterization of a non-resonant current sensor showed that in the operation range to 5 A, the sensor had a sensitivity of 0.34 V/A, non-linearity less than 1% while for a resonant current sensor in the same operation range, the sensitivity was 0.53 V/A, with a non-linearity less than 0.5%. Possible applications of ME current sensors for biomedical applications required the transition to be flexible [20] and to have nanoelectronic structures [23]. The current sensor [20] consisted of a bilayered structure based on the Cytop polymer and a magnetic tape filled with magnetically soft particles and demonstrated a possibility to realize a low-cost flexible current sensor with an improved magnetoelectric response. In [23] the fabrication of an ultra-low power current sensor utilizing a PZT-NZF nanowire array is described. Characterization of the sensor up to 70 mA showed a sensitivity of 3.24 mV/mA, a sensitivity error of 1.16%, non-linearity of 4%, a noise floor of < 2 mA, and noise density of 8.4 nA·Hz^−1/2^ at 1 kHz at the power required for a low power op amp of 0.225 mW. The contactless DC current sensor based on an ME PVDF/Metglas composite included a solenoid and the corresponding electronic instrumentation [35]. The ME sample shows a maximum resonant ME coefficient (α_33_) of 34.48 V/(cm·Oe), a linear response (R^2^ = 0.998) and a sensitivity of 6.7 mV/A. With the incorporation of a charge amplifier, an AC-RMS converter and a microcontroller the linearity is maintained (R^2^ = 0.997), the ME output voltage increases to a maximum of 2320 mV and the sensitivity rises to 476.5 mV/A. It should be noted that there was a rapid growth of works on ME current sensors and the appearance of reviews [33,34], in which extensive technological studies of the properties of permalloy films and composites of amorphous magnetic tapes and piezoelectric polymer films were carried out. These include works in which a sensor for accurate assessment of the viscosity of liquids was proposed [33] and new functional capabilities of the ME effect for the development of ultrafast, low-power and miniature electronics were considered for the example of new electronic devices such as high-speed memory, a radio frequency resonator, a compact ME-antenna, and current and weak magnetic field sensors [34]. Along with the advantages of the considered sensors, their main drawback remained, namely that associated with the need to use a bias field, which is most often created by permanent magnets. This disadvantage was corrected in [36,37,38,39,40,41] by using gradient magnetostrictive–piezoelectric structures, since in this case it is possible to obtain an internal bias field of the composite without external additional devices. In the patent [36], it is shown that a magnetostrictive-ferroelectric structure with a gradient of magnetization and polarization makes it possible to obtain internal magnetic and electric fields that replace the external bias and polarizing fields. Zhou Y. et al. considered [37] in detail the current state-of-the-art of the different self-biased structures, their working principle as well as their main characteristics. Application of self-biased structures for harvesters, memory devices and the future perspective of this research field were discussed. In [38] Mandal et al. presented the theory and the observations of ME interactions under zero bias (H_0_ = 0) in a bilayer of PZT and a ferromagnetic layer in which the magnetization was graded with the use of Ni and Metglas. At low frequencies, the ME coefficient ranged from 0.3 to 1.6 V/(cm·Oe) and depended on the thickness of the Metglas. A factor of 40 increase in the ME voltage was measured at the bending mode. Theoretical estimates of ME coefficients at low frequencies and bending modes were compared with the data. Lu C. et al. considered [39] the bending-mode ME coupling in the asymmetric laminate composite with a magnetization-graded ferromagnetic material. They developed the bending-mode resonant ME coupling model based on the proposed dynamic piezomagnetic model, the motion equation of the ME composite, and the ME equivalent circuit method. The composite structures FeCuNbSiB/Ni/PZT with negative magnetostrictive Ni and FeCuNbSiB/FeNi/PZT with positive magnetostrictive FeNi were used to confirm the validity and reliability of the theoretical model. In [40] Zhang et al. presented the results of studying the ME interaction in the structure of Terfenol-D (T), PZT-8H (P) and FeCuNbSiB (F), when the external DC magnetic field is zero. The developed theory based on the finite element method is highlighted with a direct comparison with experiments that show their close agreement. The influence of temperature on the ME effect of the T-P and F-T-P was also studied, and the results showed that with higher temperature, the ME coefficient of the structures became smaller. SrFe_12_O_19_/FeCuNbSiB/PZT self-biased ME sensor has a higher sensitivity of 198.91 mV/A at 50 Hz and the induced output voltage shows a good linear relationship to the applied 50 Hz current [42]. Also, the SrFe_12_O_19_/FeCuNbSiB/PZT sensor can distinguish small step changes of 0.01 A current. The disadvantage of a self-biased current sensor based on ME composite SFP is the inability to measure conductors with currents of different diameters (the current-carrying cable is a copper core of diameter 1.8 mm with an insulating layer of thickness 0.8 mm around the copper core).

In order to create a self-biased current sensor, in [41] the ME effect was modeled and studied in a gradient laminate structure consisting of a bidomain LN/Ni/Metglas. It was shown that the maximum value of the ME effect under bending resonance conditions was 577 V/(cm·Oe), and an optimal bias field of 3.5 Oe was generated for the structure with an 860-nm-thick Ni film. Earlier, the ME effect was investigated in a structure based on bidomain LN/Metglas in the bending vibration region [43]. Ferroelectric lithium niobate crystals possess high thermal stability of material properties (piezoelectric, elastic, electromechanical etc.) for different cuts [44], especially in transducers operating in the resonant regime [45]. Furthermore, the lead-free nature of LN meets the demands of the RoHS directive which assumes the restriction of use of certain hazardous substances in electrical and electronic equipment. Thus, applications based on LN can substitute commonly used PZT ceramics. Moreover, the magnetoelectric coefficient proportional to the ratio of d/ε [46] and low piezoelectric coefficients of LN crystals compensate by also having low dielectric permittivity and this ratio can be higher for optimal crystal cut of LN than in PZT ceramics. In most used commercial applications y + 128°-cut LN ratio of d/ε = 0.54 pm/V [43] is very competitive in comparison with most used PZT ceramics (#APC851) where d/ε = 0.1 pm/V [46]. From a practical point of view, ME composites for magnetic or current sensors should have high sensitivity to magnetic fields at low frequency, compact size, and low power consumption. ME composites based on bidomain LN crystals are very suitable for this purpose. An additional advantage of lithium niobate crystals is the absence of a lead component in their composition. As is known, the bidomain LN crystals demonstrated excellent properties in the application of magnetoelectric (ME) magnetic sensors [43,47], vibration sensors [48] and energy harvesters [49].

An analysis of investigations on the study of gradient magnetostrictive-piezoelectric structures shows that, despite the large amount of information obtained on ME sensors, there remains a need for detailed calculation of the internal magnetic fields in the gradient structure, the calculation of frequency-dependent ME coefficients and finding a linear section on the operating characteristic of the sensor in a given range of working currents. This article is dedicated to solving these problems based on the consideration of the promising structure of the bidomain LN/Ni/Metglas.

## 2. Samples

**Figure 1:**
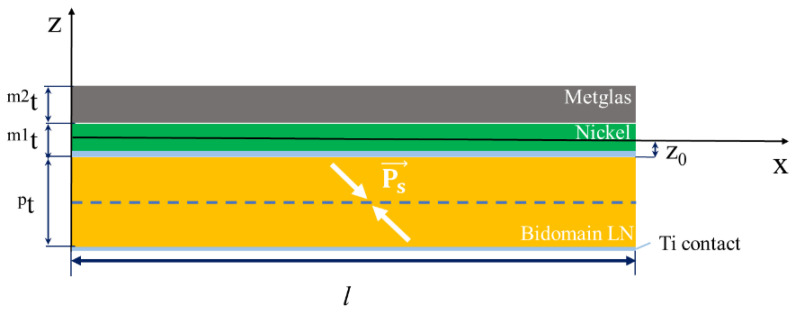
Schematic view of the magnetoelectric (ME) gradient structure.

Samples of ME gradient structure contained layers of bidomain LN y + 128°-cut/Ni/Metglas. A scheme of the ME gradient structure is presented in Figure 1. The reference sample used an ME composite structure without an Ni layer (bidomain LN y + 128°-cut/Metglas). Samples of bidomain LN for gradient ME composites were produced from commercial single domain crystals with y + 128°-cut congruent LN by using the diffusion annealing process [48]. The bidomain structure has two macrodomains with opposite spontaneous polarization vectors (“head-to-head”) and a narrow polydomain area or saw-shaped domain wall in the middle of the plate [50].The six equal bidomain LN samples with a length *l* = 20 mm, thickness ${}^{p}t$ = 0.5 mm and width *s* = 5 mm were prepared.

The Ti electrodes were deposited on both faces of the LN crystals by DC magnetron sputtering (Sunpla 40TM). The Ti electrode was also used as a cathode for Ni electrochemical deposition. The Ni layers were deposited on the ground face of the bidomain LN samples. The electrochemical deposition process was carried out in a homemade setup which included a power supply and a bath for electrodepositing nickel comprising a solution of nickel sulphate, boric acid, and distilled water [51]. A pure nickel plate was used as the anode, the deposition temperature was 75 °C, and the current between the anode and cathode was 5 mA during the process. The resultant thicknesses tm1 of the deposited Ni layers were 2, 3, 4, 5, 7 and 10 μm. The deposition rate of the Ni was ≈0.3 μm/min. The thickness of the Ni layers was controlled by a profilometer (Alpha-Step IQ).

**Figure 2:**
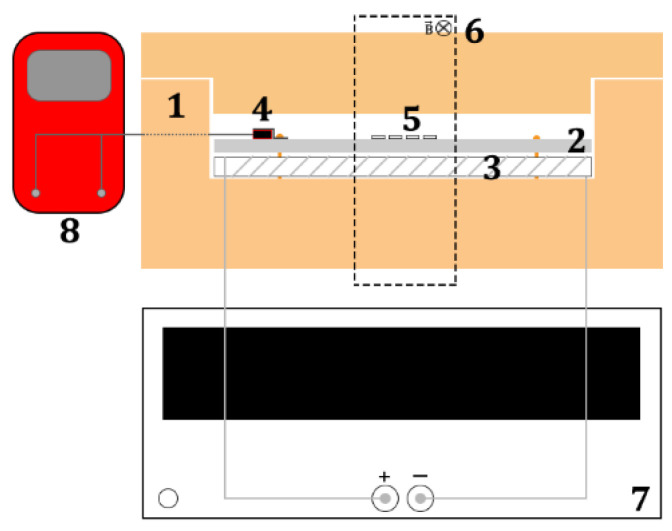
Schematic representation of the setup for annealing samples of bidomain LN y + 128°-cut/Ni in the magnetic field.

In the next step composite samples of bidomain LN y + 128°-cut/Ni were annealed in the magnetic field. The annealing temperature was 400 °C with an exposure time of 1 min. This temperature was chosen because it is above the Curie temperature ($T_{C}$ = 358 °C [52,53,54]), which allows orienting the magnetization of the magnetic domains of the Ni layer along the length of the ME sample in an external magnetic field. The magnitude of the external magnetic field was 1000 Oe. The annealing in the magnetic field was conducted in a homemade setup which included: 1 a fireclay furnace, 2 an aluminum plate, 3 a nichrome wire heater, 4 a temperature sensor Pt1000 (MN222, class B), 5 ME samples, 6 the dotted line indicates the magnetic field area which was applied by permanent magnets, 7 a power supply which applied current on nichrome wire and 8 a multimeter was controlled and measured resistance of temperature sensor. The block scheme of the annealing setup is presented in Figure 2.

Gradient ME composites were subsequently prepared by bonding a single layer of commercial 2826 MB type Metglas foil (Hitachi Metals Europe GmbH), with a thickness tm2 = 29 μm, on the Ni layers. The bonding was achieved using an epoxy adhesive (Devcon epoxy 14260) which was then cured at 50 °C for 3 h under an applied vertical pressure of 100 kPa by a piston cylinder.

## 3. Theoretical and Experimental Approach

**Figure 3:**
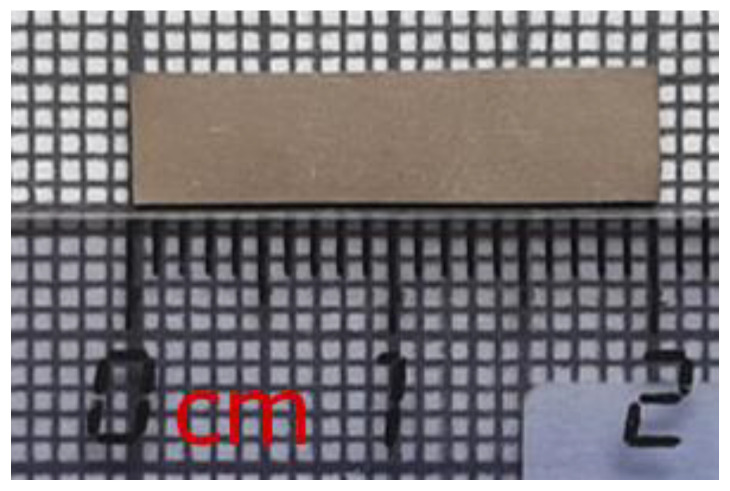
Photo of one of the investigated structures based on bidomain LN/Ni/Metglas.

**Figure 4:**
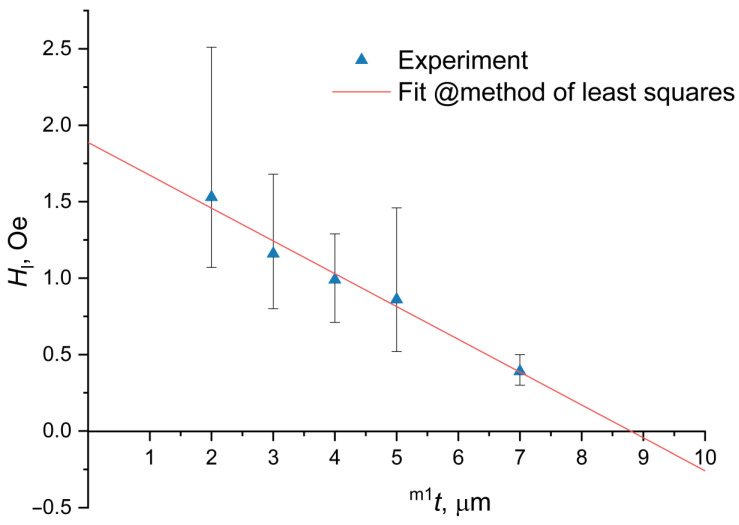
Dependence of the magnitude of the external magnetic field with error bars, at which point the linear section begins, on the nickel thickness.

When using one magnetostrictive phase (Metglas) in an ME current sensor, it is necessary to use a biased field to shift the initial operating point of the sensor to the beginning of the linear section of the dependence of the output voltage on the measured current. The use of a gradient magnetostrictive phase, in which, in addition to Metglas, nickel is also present, makes it possible to obtain an internal magnetic field in it. This internal field can be used instead of an external biased field. This makes it possible to shift the starting operating point towards the beginning of the linear section without using an external source of a biased field. For the development of a contactless ME current sensor based on bidomain LN/Ni/Metglas, theoretical and experimental studies were carried out. The appearance of one of the structures is shown in Figure 3. To enter a linear section as shown in Figure 4, using an internal bias field, we investigated gradient structures based on a bidomain LN y + 128°-cut with various thicknesses of the Ni layer indicated in Table 1.

### 3.1. Internal Fields in a Gradient Two-Layer Magnetostrictive Structure Nickel/Metglas 2826 MB

It was shown in Appendix A.1 that if the thickness of the nickel layer tm1 (the m1 index indicates that it is the magnetostrictive nickel layer), the Metglas layer tm2 (the m2 index indicates that it is the magnetostrictive Metglas layer) and the remnant magnetization of nickel $B_{r1}$, then an internal magnetic field appears in the gradient magnetostrictive structure, which is equivalent to an external field at a sufficient distance from the structure:

(1) H0=Br1tm1μ0tm2.

It is obvious that the value of the external magnetic field $H_{l}$, at which the linear section of the dependence of the ME voltage coefficient on the constant bias field begins, decreases by the value $H_{0}$ for an ME composite with a nickel thickness tm1 in comparison with the same ME composite, only without nickel layer. The dependencies of the ME voltage coefficient on the external magnetic field were obtained experimentally for the following nickel thicknesses: 2, 3, 4, 5, 7, 10 μm and the beginning of a linear section was found on each dependence. Figure 4 shows the points with error bars of the experimental dependence $H_{l}$ on the nickel thickness tm1 obtained in this way. The external magnetic field magnitude at which the linear part begins can be defined for every sample with a certain thickness of Ni layer by this method. The points of the experimental dependence of the resonance value of the ME voltage coefficient vs. external magnetic field were taken, starting from the first one (0 Oe), up to a certain value where the end of the linear section is assumed. Linear regression was made and linearity error R^2^ was calculated for the set of points with this experimental dependence. Next, the first point was deleted from the data and the linearity error R^2^ was calculated for this new group of points. This process was repeated for every new step. Since at the beginning of the experimental dependence there is a nonlinear dependence similar to a quadratic one, then with a gradual removal of points (corresponding to small values of a constant magnetic field) the linearity error R^2^ grows rather quickly, but after a certain point the change in R^2^ becomes insignificant. This point is considered to be the beginning of the linear part. For this point error bars were calculated as the difference between this point and previous and next ones on this dependence. A straight line was drawn through the experimental points by the least squares method:

(2) Hl=k1tm1+k2,

where the coefficients of the straight line were:

(3) $$\begin{array}{l} k_{1}=-0.2615\\ k_{2}=2.3769 \end{array}$$

The residual induction of nickel was determined via $k_{1}$:

(4) Br1=−k1μ0tm2=0.00062 T.

### 3.2. Flexural Mode of the ME Effect in the Gradient Structure of Bidomain LN/Ni/Metglas

The formula for the ME voltage coefficient in the EMR region of the bending mode for the gradient structure of bidomain LN/Ni/Metglas with freely fixed ends is given by the following expression:

(5) αE=2tm2tp2〈q11〉〈h31〉〈β33S〉(r1r4+r2r3−r2−r4)t[〈c11〉klt3〈β33S〉(1−r1r3)−2tp3〈h31〉2(r1r4+r2r3−r2−r4)].

All quantities included in (5) are described in detail in Appendix A.2. In deriving (5), following [55,56], the ME voltage coefficient was calculated based on the effective electric field strength obtained by dividing the electric voltage across the piezoelectric phase by the total thickness of the composite *t*. If the ME composite did not contain nickel, then the pseudo-piezomagnetic coefficient of the Metglas would be given by the formula [39]:

(6) q211=2λs(coth(ηH)−1ηH)[η(1−coth2(ηH))+1ηH2],

where $\lambda_{s}=12\cdot 10^{-6}$ is the saturation magnetostriction of the Metglas [57],

(7) $$\eta =\frac{3\chi_{m}}{M_{s}},$$

and where $\chi_{m}$ and $M_{s}$ are the initial magnetic susceptibility and saturation magnetization of the Metglas, respectively.

If the nickel layer thickness in the ME composite is tm1, then the external field H in (6) should be replaced by (H+Br1tm1μ0tm2).

**Figure 5:**
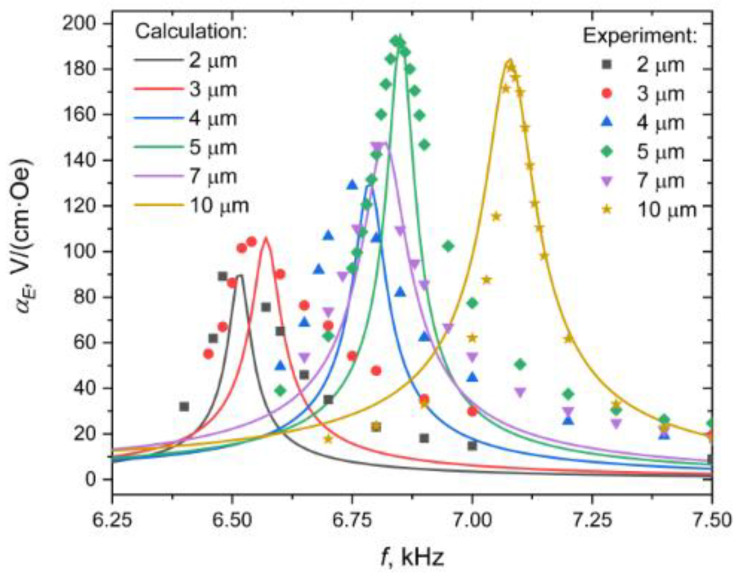
Dependence of the ME voltage coefficient on the frequency of an alternating magnetic field for samples with different nickel layer thicknesses at zero bias field. Points are experimental data, solid lines are theoretical dependencies. When the experimental dependencies were taken, the amplitude of the alternating magnetic field was 1 Oe.

Figure 5 shows the dependencies of the ME voltage coefficient on the frequency of the alternating magnetic field for samples with different thicknesses of nickel layer at zero bias field. To take into account losses in the calculation, it is assumed $\omega =2\pi \left(1+\frac{1}{2Q}i\right)f$, where Q is the quality factor of the resonance. This figure also shows the corresponding experimental data.

It is seen that the calculated curves are in satisfactory agreement with the experimental data. Some discrepancy between the theoretical curves and the experimental points is apparently due to the effect of the adhesive layer between Ni and Metglas, which was not considered in the theory. There is also a noticeable tendency that the thicker the nickel layer in the sample, the bigger the value of the resonance maximum of the frequency dependence of the ME voltage coefficient. This corresponds to our theoretical ideas that the bigger the thickness of nickel layer in the sample, the greater the magnitude of the internal field arising in it, and the stronger the field dependence of the ME voltage coefficient shifts to the left.

From (5) for the resonant frequency of the alternating magnetic field, the following expression is obtained for the resonance value of the ME voltage coefficient:

(8) αE=−2Qtm2tp2〈q11〉〈h31〉〈β33S〉〈c11〉t4〈β33S〉+〈h31〉2tp3t.

**Figure 6:**
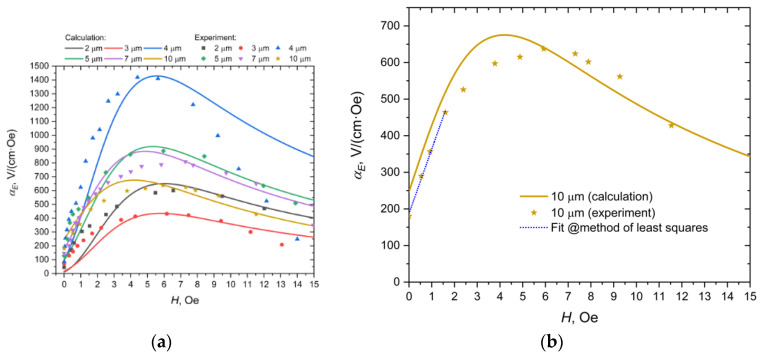
(**a**) Dependence of the resonance maximum of the ME voltage coefficient on the magnitude of the biased field for samples with different thicknesses of nickel. Individual points are experimental data, solid lines are theoretical dependencies. (**b**) Dependence of the resonance maximum of the ME voltage coefficient on the magnitude of the biased field for samples with thicknesses of nickel of 10 μm. Asterisks are experimental points and solid lines show the theoretical dependence. The dashed blue line is drawn through the first 4 experimental points using the least squares method.

Figure 6 shows the measured and calculated values using Equation (8) dependencies of the resonance maximum of the ME voltage coefficient on the external magnetic field for samples with different nickel layer thicknesses.

As seen from Figure 6a, the theoretical dependencies are in satisfactory agreement with the experimental data. The maxima of the field dependencies are quite different for different samples, which, apparently, is associated with different technology and, accordingly, quality factors. In Figure 6b, it can be seen that for a sample with a nickel thickness of 10 μm, the linear section starts from zero value of the constant external magnetic field and continues up to 1.6 Oe. This is consistent with the graph in Figure 4.

Thus, the internal field created by nickel in the Metglas completely replaces the need for an external source of constant magnetic field in the design of the current sensor to reach the linear section. Therefore, this particular sample with a nickel thickness of 10 μm was chosen for use in a DC ME sensor.

## 4. Principle of Operation and Design of ME Current Sensor

**Figure 7:**
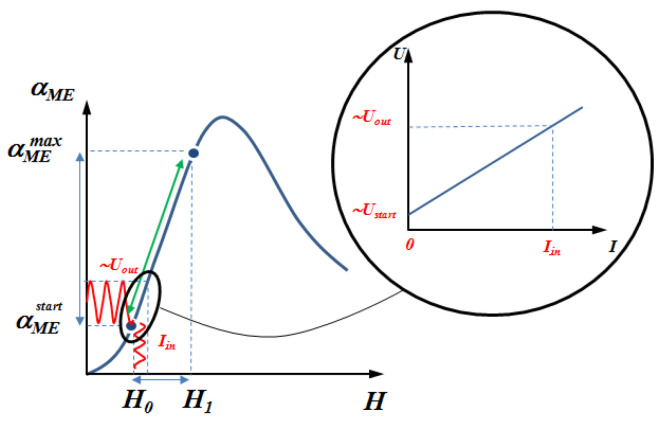
The operating principle of the ME current sensor.

Earlier in the article [32], an ME current sensor was presented, the sensitive element of which is the magnetostrictive-piezoelectric structure Metglas/PZT/Metglas. To ensure high linearity of the sensor, it was necessary to use a bias field (in our case it was a small permanent magnet) to reach the linear section as showed in Figure 7. The presented ME current sensor in this article works according to the same principle as the previously developed current sensor, however, a significant difference is the sensitive element of the device. The sensing element of the new current sensor is a magnetostrictive-piezoelectric structure consisting of bidomain LN *y* + 128°-cut/Ni/Metglas 2826 MB with 10 μm of Ni layer. This gradient structure has an internal bias field that allows one to use a linear section of the operating characteristic without a permanent magnet and to achieve the self-biased current sensor.

**Figure 8:**
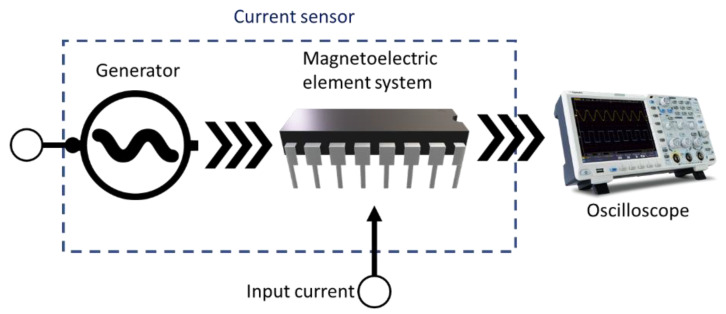
The block scheme of the ME current sensor.

An ME current sensor consists of a generator; the ME element system is presented in Figure 8. An ME element system consists of a sensitivity element and inductive coil and converts the energy of the alternating magnetic field of the coil proportional to the amount of current flowing in electrical voltage.

**Figure 9:**
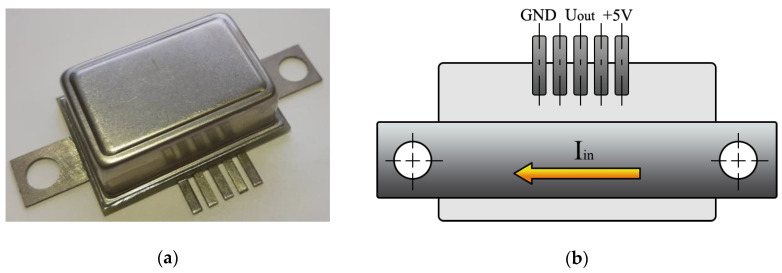
(**a**) Designed body for the ME current sensor and (**b**) schematic description of the ME current sensor.

Figure 9 shows the body for ME current sensor. The sensing element is an asymmetric structure operating in bending mode regime with a central point fastening with free ends of the composite and installed in the inductance coil. When a direct current was flowing through the conductor, the output signal was recorded on an oscilloscope using electrodes. The directions of the external magnetic field connected with incoming current I_in_ are shown in Figure 9b; the alternating magnetic fields are parallel to each other. A current bus with the indicated direction of measured current was used as a conductor (Figure 9b). Reliable measurement of current is only possible with the sensor’s correct connection. Mounting of the sensor could be made by any convenient method, i.e., adhesive, surface mounting (SMD) etc.

**Figure 10:**
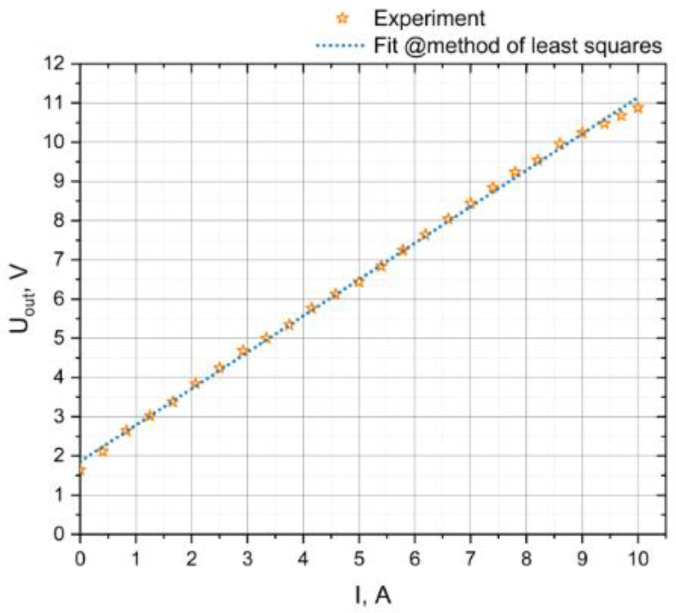
Output voltage versus measured current plot.

Figure 10 shows a graph of the output dependence of the ME current sensor. When the output dependence was taken, the amplitude of the alternating magnetic field was 0.2 Oe. The sensor has a fairly good linearity; the linearity is maintained at 99.8%. The sensitivity is 0.9 V/A and the current consumption is no more than 2.5 mA. The range of the measured current was limited by the available equipment. The zero value of the measured current in Figure 10 corresponds to the zero value of the external constant magnetic field in Figure 6b, and the attained measured current value of 10 A corresponds to a magnetic field value of 1.1 Oe. Since the linear section in Figure 6b continues up to 1.6 Oe, this means that the potential DC measurement range of this sensor is even wider than up to 10 A.

## 5. Discussion

It is of interest to compare the characteristics of the known current sensors and the developed sensor. For this purpose, we compiled a table of comparative characteristics of ME current sensors and a Hall effect current sensor CSLW6B5 from the American corporation Honeywell International, Inc., Charlotte, NC, USA.

Current sensors based on the ME effect operate on a similar principle of operation as shown in Figure 7. As a result, an analysis of the characteristics of Table 2 shows that the proposed sensor has higher sensitivity and lower power consumption compared to both ME sensors and the Hall sensor and is ready for practical use in a wide range of devices. An additional advantage is also the absence of lead (lead-free) in the sensor structure.

It is of interest to discuss the methods for calculating self-biased structures that are known from publications of other authors [38,39,40]. In contrast to work [38], in which the authors use the hypothesis that the electric field strength is independent of the thickness coordinate *z*, when calculating the flexural mode of the ME effect, in this paper, as in [39], we used a more justified ratio for the size ratio of the investigated ME composite. Our hypothesis is based on the independence of the electric induction from the thickness coordinate *z*. This made it possible to obtain a more accurate expression for the ME stress coefficient for the flexural mode of the ME effect.

In [38], the authors find the residual magnetizations in two magnetostrictive phases of the gradient structure using the free energy density of the gradient magnetostrictive structure, considering that the initial magnetizing field is directed perpendicular to the plane of the ME composite. In this article, the method of magnetic circuits is used to find the internal magnetic fields in the case when the initial bias field is directed along the length of the ME composite. Such internal magnetic fields directed along the length of the ME composite make it possible to achieve significantly higher values of the ME voltage coefficient, since there is no need to act against the demagnetizing factor of the shape of the thin plate.

In [39], the authors use the method of finite element modeling to find internal magnetic fields in gradient magnetostrictive structures. The finite element modeling method allows obtaining only specific numerical results for given sizes of magnetostrictive phases. In this article, to find the internal magnetic fields, the magnetic circuit method is used, which allows one to obtain analytical dependencies of the internal magnetic fields on the thicknesses of the magnetostrictive phases. Using these dependencies, on the basis of a certain amount of experimental data for several thicknesses of magnetostrictive phases, it is possible to predict the results for other thicknesses.

In [40], the authors use the finite element modeling method to fully calculate the self-bias of the ME effect in the bending mode. The finite element modeling method allows one to obtain only specific numerical results for the specified phase sizes of the ME composite. In our work, both parts of the calculation model are analytical, which allows us to further adapt this model for other materials and sizes of ME composites.

## 6. Conclusions

The article discusses the practical possibility of using the gradient structure of bidomain LN/Ni/Metglas as a sensitive element of the ME current sensor. With the aim of a deeper understanding of the operation of the gradient structure, the article considers in detail the calculations of the internal magnetic fields of the ME structure and the ME voltage coefficient for the bending mode and the method for determining the linear section on the operating characteristic of the current sensor. For the investigated gradient structures with a nickel thickness of 2, 3, 4, 5, 7, and 10 μm, theoretical and experimental dependencies of the ME voltage coefficient on the frequency of the AC magnetic field and the magnitude of the external biased field were measured. The results obtained made it possible to determine the linear section in the range from 0 to 10 A on the working characteristic of the current sensor. The studies have shown that the optimal structure for the self-biased current sensor with the required linear section is Ni with a thickness of 10 μm. The proposed contactless ME current sensor worked in the bending mode and its characteristics are as follows: a measured current range up to 10 A; a sensitivity of 0.9 V/A; the current consumption is not to exceed 2.5 mA, and the linearity is to be maintained at 99.8%.

## Figures and Tables

**Table 1:** Structure and geometric dimensions of the samples under study.

Structure	Plate LN, mm	Thickness Ni, μm	Thickness Metglas, μm
Ti(100 nm)/LN/Ti(100 nm)/Ni/Metglas2826 MB	20 × 5 × 0.5	2	29
		3	
		4	
		5	
		7	
		10	

**Table 2:** Comparative of characteristics of current sensors.

Sensors	Measuring Principle	Primary Current, Measuring Range, I_pm_ (A)	Sensitivity (V/A)	Supply Voltage (V)	Linearity (R^2^∙100), %	Output Voltage Range U_out_ (V)	Size (mm)	References
Magnetoelectric sensor	Magnetoelectric effect	0–10	0.9	20 ± 5%	99.8	1.64–10.88	30 × 20 × 10	This paper
CSLW6B5	Hall effect	±5	0.2	4.5–10.5	98.9	2.7–3.7	16 × 14 × 10	-
Magnetoelectric sensor (Metglas/PZT/Metglas)	Magnetoelectric effect	0–5	0.53	5 ± 10%	-	1.5–4.5	30 × 20 × 10	[32]
Magnetoelectric sensor (Metglas/PVDF)	Magnetoelectric effect	0–5	0.48	-	99.7	0–2.32	-	[35]
Magnetoelectric sensor(SrFe_12_O_19_/FeCuNbSiB/PZT)	Magnetoelectric effect	0–5	0.2	-	99.9	0–1	-	[42]

**Figure A1:**
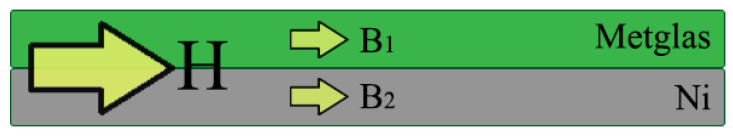
Intrinsic magnetic fields in the bilayer magnetostrictive structure Ni/Metglas.

## Appendix A

### Appendix A.1. Intrinsic Magnetic Fields in Gradient Bilayer Magnetostrictive Structure Ni/Metglas 2826MB

The structure should be separately considered for finding intrinsic magnetic fields in gradient bilayer magnetostrictive structure Ni/Metglas shown in Figure A1.

A magnitude of magnetic induction in outer space while measuring a dependence of Metglas 2826 MB magnetostrictive coefficient from the value of the extrinsic field $H_{0}$ is:

(A1) $$B_{2}=\mu_{0}H_{0}.$$

In case of continuity of magnetic induction’s normal component, its magnitude inside Metglas is the same.

As shown on the demagnetization curve of Metglas 2826MB [57], the magnetic induction values that we need ($H_{0}\leq 15$ Oe) correspond to very small values of the intrinsic field in Metglas H<0.05 Oe. In that case the magnetic induction is mostly defined by magnetization of Metglas.

First, the composite should be magnetized by a powerful longitudinal external field until the moment that nickel and Metglas reach full saturation. After that, the external field should be slowly removed. In this case, Metglas partially remagnetizes in the opposite direction, and nickel obtains residual magnetic induction. Magnetic field intensities inside Ni and Metglas would be the same (H<0.05 Oe) because the tangential component of the magnetic field intensity should be continual.

The magnetic induction of Ni is then: $B_{1}=B_{r1}$, where $B_{r1}$ is the residual magnetization of Ni.

The total magnetic flux through the composite should be equal to zero because of the absence of the external field and due to the continuity of magnetic flux:

(A2) −B2tm2s+Br1tm1s=0.

From Equation (A2)

(A3) B2=Br1tm1tm2.

Equation (1) is made from (A1) and (A3).

The value of the pseudo-piezomagnetic coefficient for Ni with |H|<0.05 is q111=−1.9⋅10−9 m∙A^−1^.

### Appendix A.2. Bending Mode of ME Effect in Gradient Structure Metglas/Ni/bidomain LN

The full thickness of the piezoelectric is:

(A4) tp=tp1+tp2.

The full thickness of the gradient magnetostrictive phase is:

(A5) tm=tm1+tm2.

The full thickness of the composite is:

(A6) t=tp+tm.

The volume parts of the piezoelectrics and ferromagnetics are:

(A7) νp1=νp2=tp1t, νm1=tm1t, νm2=tm2t.

The effective density of the composite is:

(A8) ρ=(νp1+νp2)ρp+νm1ρm1+νm2ρm2,

where ρp, ρm1 и ρm2 are the densities of LN, Ni and Metglas, respectively.

(A9) c¯11D=(sp11E−dp1312ε33Tε0)−1h¯p131=c¯11Ddp131ε33Tε0h¯p231=c¯11Ddp231ε33Tε0β¯33S=1+h¯p131dp131ε33Tε0,

where sp11E is the longitudinal compliance of LN, dp231=−dp131 are the piezoelectric coefficients of the bidomain LN layers, $\varepsilon_{33}^{T}$ is the relative dielectric permeability of LN.

(A10) q¯111=Ym1Bq111Ym1B=Ym11−Km1112Km1112=Ym1q1112μ1μ0,

where Ym1, q111 and μ1 are Young’s modulus, the pseudo-piezomagnetic coefficient and the relative magnetic permeability of Ni, respectively.

(A11) Ym2B=Ym21−Km2112Km2112=Ym2q2112μ2μ0q¯211=Ym2Bq211,

where Ym2, q211 and μ2 are Young’s modulus, the pseudo-piezomagnetic coefficient and the relative magnetic permeability of Metglas, respectively.

(A12) 〈h31〉=14h¯p131〈q11〉=q¯111tm1(2z0+tm1)+q¯211tm2(tm2+2z0+2tm1)2tm2.

The position of the divide border between LN and Ni relative to the neutral line is:

(A13) z0=c¯11Dpt2−Ym1Bm1t2−2Ym2Bm1tm2t−Ym2Bm2t22(Ym1Bm1t+Ym2Bm2t+c¯11Dpt).

(A14) 〈c11〉=1t3(D−tp3〈h31〉2β¯33S).

The full cylindrical stiffness of the ME composite is:

(A15) D=Dp+Dm1+Dm2,

where

(A16) Dp=13c¯11Dtp(tp2−3tpz0+3z02)Dm1=13Y˜m1tm1(tm12+3tm1z0+3z02)Dm2=13Y˜m2tm2(tm22+3tm2(z0+tm1)+3(z0+tm1)2).

(A17) $$k={\left(\frac{\rho }{t^{2}\langle c_{11}\rangle }\omega^{2}\right)}^{\frac{1}{4}},$$

(A18) $$r_{1}=\cosh\left(kl\right),\;r_{2}=\sinh\left(kl\right),\;r_{3}=\cos\left(kl\right),\;r_{4}=\sin\left(kl\right).$$

When calculating the theoretical dependencies, the following values of material constants for Ni were used: ρm1=8902 kg m^−3^, Ym1=5.0⋅1010 N m^−2^, μ1=300; for Metglas 2826MB: ρm2=7900 kg m^−3^, Ym2=1.0⋅1011 N m^−2^, μ2=1.0⋅104; for LN: ρp=4647 kg m^−3^, sp11E=6.91⋅10−12 m^2^ N^−1^, $\varepsilon_{33}^{T}=49.8$, dp131=−dp231=−27.24⋅10−12 m V^−1^, and the thicknesses of bidomain LN layers are tp1=tp2=2.5⋅10−4 m.
